# Age-related differences in integrin expression in peripheral blood lymphocytes

**DOI:** 10.1186/1742-4933-7-5

**Published:** 2010-04-26

**Authors:** Christine V Crooks, Martin L Cross, Clare R Wall

**Affiliations:** 1 Institute Food, Nutrition and Human Health, Massey University, Auckland, New Zealand; 2 Landcare Research/Manaaki Whenua, Lincoln, New Zealand; 3 School of Medical Sciences, University of Auckland, Auckland, New Zealand; 4 Department of Virology and Immunology, LabPlus, Auckland Hospital, Auckland 1023, New Zealand

## Abstract

Alpha integrins play an important role in cell to cell and cell to extra-cellular matrix interactions required for an effective T-lymphocyte-mediated immune response, however little is known about age related differences in expression of alpha integrins on T-cells in humans. We here measured alpha-4 (α4) integrin (CD49d) expression on T-lymphocytes via peripheral blood sampling, comparing parameters between cohorts of young and old adults. No age-related differences were found for the absolute numbers of T-cells, although the percentage of CD4+ T-cells in older adults was significantly greater and the percentage of CD8+ T-cells lower than in younger cohorts. Percentage and absolute numbers of CD3+ T-cells co-expressing CD49d were significantly lower in older adults compared to younger cohorts, and the percentage of gated CD4+ and CD8+ cells that co-labelled positively for CD49d was also reduced in this group. There were no age-related differences in circulating levels of cytokines (Type I interferons) that are known to regulate cell surface integrin expression. Reduced expression of alpha integrins on T-cells may be an early indicator of the loss of homeostatic control that occurs with ageing, contributing to diminished effector T-cell responses during senescence.

## 

The ageing process leads to marked changes in the composition, function and competence of the human immune system, commonly termed immunosenescence [1,2]. As a consequence of an altered immune system, older populations experience increased morbidity and mortality from respiratory tract pathogens [3], an increase in gastrointestinal infections [4], and antigen specific responses are diminished with orally administered vaccines [5]. Age-related phenotypic and functional changes to the T-cell component of adaptive immunity occur [6] while B-cell function and the innate immune system are less affected [7,8].

An effective immune response depends on T-cells being available to respond to low levels of peptide antigen following interaction with antigen presenting cells (APCs) [8,9]. Naïve T-cells constantly move through lymph nodes via high endothelial venules (HEVs) which ensure their exposure to antigens in an environment conducive to optimal stimulation [10]. The necessary cellular and cell to extra cellular matrix interactions are mediated by a large family of αβ-integrins, in particular the α4 integrins (α4β1 and α4β7), which direct peri- and extra-vascular lymphocyte trafficking [9,11,12]. It is known that expression levels of alpha-integrins can be modified by a number of endogenous signals, including the localised effect of cytokines such as interferons [13,14] and biochemical abnormalities [15].

Overall T-cell numbers are maintained until the 7^th ^decade of life in older adults [8,16]. However, age-related thymic involution [7] and T-cell functional changes occur [17], including a reduced T-cell receptor (TCR) repertoire [18], and a reduced pool of naïve (CD45RA^hi^) cells relative to memory (CD45RO^hi^) cells [2]. Reduced cell trafficking has also been observed for CD4 T-cells (via down-regulation of CD31 which is involved in T-cell adhesion and activation) in older humans [2,19], for peripheral blood mononuclear cells in old rats [20], and for T-cells in old mice [21]. Ageing also reduces α4β7 expression on peripheral blood lymphocytes in rats [20], although a similar phenomenon has not been recorded in humans. Current evidence for age-related differences in lymphocyte expression of α4 integrins is inconsistent: one study has reported an increase in CD49d expression on T-cells overall, but with decreased expression in subjects who express normal CD4:CD8 T-cell ratios [22], while in another study [17] no age-related differences for CD49d-expressing T-cell populations were reported (although a higher level of density expression of cell surface of CD49d was reported in this latter study).

Here, we have used cell surface labelling and flow cytometric analysis of peripheral blood T lymphocyte populations to compare the relative expression of α4 integrins between different adult age groups. We present differences in the percent and numbers of α4 integrin-expressing lymphocytes in the circulating blood of older adults in comparison to the same parameters in the blood of young adults. Via numerical comparison we also present the differences in the proportion of cells co-expressing alpha integrin for each T-cell subset observed.

Thirty adults volunteered and 29 completed the study requirements. There were ten young non-exercising and nine exercising adult controls (there is considerable exercise-induced redistribution of T-cells within lymphoid and non-lymphoid organs in athletes [23]). Ten older adults were the test group (Table 1). Blood was sampled by venepuncture from the antecubital vein. The general health of all participants was determined by measurement of plasma albumin and blood ferritin levels (which were within reference range values for adults [Table 1]), and C-reactive protein levels (50% young non-exercising and 20% older adults were above the reference level [24]). Additionally, liver function enzymes were measured in the older adults as an indicator of any potential underlying pathology ([24,25]); these were generally within reference range values for adults [Table 1]). The study protocol was given ethical approval by the Auckland Ethics Committee, Ministry of Health, New Zealand (AKX/03/07/182) and (AKY/04/10/268).

**Table 1:** Participant characteristics for young non-exercising, young exercising and older adults. Age and body mass are expressed as mean ± SEM (range), and general health parameters (plasma ferritin, albumin, and C-reactive protein [CRP]) are expressed as mean ± SEM

Parameter	Young adults		Older adults n = 10
	Non-exercising n = 10	Exercising n = 9	
Gender (n)			
Female	6	6	5
Male	4	3	5
Age (years)	22.7 ± 0.73 (21 - 26)	18.4 ± 0.68 (17 - 22)	71.2 ± 0.52 (69 - 76)
			
Body mass (Kg)			
Female	59.7 ± 4.5 (49.6 - 77.6)	73.7 ± 5.7 (61.0 - 98.1)	58.4 ± 2.9 (50.0 - 68.0)
Male	73.2 ± 6.0 (61.0 - 89.7)	80.8 ± 5.7 (70.8 - 90.7)	76.5 ± 2.8 (65.9 82.3)
*Ferritin (μg/L)	76.2 ± 19.0	72.8 ± 26.5	185.8 ± 16.6
*Reference range*	*F = 20-160, M = 20-250 *[24]		*F = 20-280, M 20-450 *[25]
Albumin (g/L)	43.0 ± 0.8	43.1 ± 0.4	42.5 ± 0.3
*Adult reference range = 35-45 *[24]			
CRP (mg/L)	6.1 ± 0.9	3.6 ± 0.3	7.6 ± 2.2
*Adult reference range = 0-5 *[24]			

F = Female, M = Male

Liver function enzyme levels for the older adults (with adult reference ranges in parentheses) were:

84 ± 20 U/L (40-130) for alkaline phosphatase; 25 ± 7 U/L (<40) for aspartate transaminase; 22 ± 7 U/L (<25) for alanine transaminase.

Four fluorochromes were used to facilitate the identification of lymphocyte populations in peripheral blood. Labeling with various combinations of a monoclonal antibody matrix allowed gating of CD45+ lymphocytes co-expressing CD3+ (T-cells). The surface markers were interchanged to allow further phenotypic analysis of CD4 and CD8 cells and CD49d expression. Cell surface markers analysed were CD3 [FITC] (Becton Dickson [BD] Cat. No. 349201), CD45 [PERCP] (BD.Cat. No. 347464), CD3 [APC] (BD.Cat. No. 340440), CD4 [FITC] (BD.Cat. No. 340133), CD8 [FITC] (BD.Cat. No. 347313) and CD49d [PE] (Pharmingen, Cat.No.555503), Isotype negative control was Pharmingen, Cat.No.555749. FACS analysis was performed on a FACScalibur flow cytometer (Becton Dickson) and analysed using Delphic software. Absolute numbers of cells expressing a phenotype were determined by multiplying the relative percentage of the lymphocyte subset by the absolute number of lymphocytes in the same blood sample. Additionally, levels of interferon (IFN)-α and IFN-β2 were assessed in plasma samples prepared from peripheral blood, using capture ELISA kits as per the manufacturer's instructions (R&D Systems Ltd, Minneapolis, USA). Assays had lower limits of sensitivity of 25.0 and 2.0 pg cytokine/mL, respectively.

Data sets were assessed for the percentage and absolute number of cells expressing a phenotype by two-way analysis of variance (SAS Release 8.02, SAS Institute, Inc, Cary, USA); the same analysis was applied to plasma interferon levels. Percentages and absolute numbers of T-cells (CD3+) did not differ between the study groups (Table 2). The older adults had proportionally more CD4+ T-cells compared to the younger cohorts (*P *= 0.011), but due to the low overall lymphocyte counts in this group, absolute CD4+ T-cell counts per L of blood did not differ between cohorts. In contrast, both the percentages (*P *= 0.026) and absolute numbers (*P *= 0.024) of CD8+ T-cells were significantly lower in the older adults compared to the younger cohorts (Table 2).

**Table 2:** Flow cytometry data (mean ± SEM) for gated lymphocytes

Parameter	Young adults		Older adults	*P*
Percentage of gated lymphocytes expressing a phenotype	Non-exercising	Exercising		
CD3	71.40 ± 1.40	76.7 ± 1.75	70.4 ± 1.85	*0.152*
CD3/CD49d	65.2 ± 1.52	71.4 ± 2.21	53.0 ± 3.24	<*0.001*
CD3/CD4	42.95 ± 1.37	42.87 ± 1.58	51.8 ± 2.99	*0.011*
CD3/CD4/CD49d	37.1 ± 1.32	38.0 ± 1.38	36.8 ± 2.92	*0.747*
CD3/CD8	23.7 ± 1.53	23.2 ± 2.41	16.4 ± 2.03	*0.026*
CD3/CD8/CD49d	23.2 ± 1.47	23.3 ± 2.19	15.6 ± 2.06	*0.010*
				
Overall counts of gated lymphocytes (× 10^9^/L)	2.19 ± 0.15	2.07 ± 0.15	1.69 ± 0.18	*0.673*
				
Absolute numbers of gated lymphocytes expressing a phenotype (× 10^9^/L)				
CD3	1.54 ± 0.09	1.58 ± 0.06	1.27 ± 0.17	*0.189*
CD3/CD49d	1.40 ± 0.08	1.47 ± 0.09	0.98 ± 0.14	*0.008*
CD3/CD4	0.92 ± 0.06	0.89 ± 0.06	0.84 ± 0.09	*0.674*
CD3/CD4/CD49d	0.80 ± 0.05	0.78 ± 0.05	0.66 ± 0.66	*0.388*
CD3/CD8	0.52 ± 0.05	0.48 ± 0.06	0.30 ± 0.06	*0.024*
CD3/CD8/CD49d	0.51 ± 0.05	0.48 ± 0.5	0.28 ± 0.06	*0.016*

SEM = The standard error of the mean.

Both the percentage (*P *< 0.001) and absolute numbers (*P *= 0.008) of T-cells co-expressing CD49d were significantly lower in the older adults compared to the younger cohorts (Table 2). When considering CD49d expression by T-cell sub-sets, percentages and absolute numbers of CD4+ cells co-expressing CD49d were lowest in the older cohort (although these differences were not statistically significant in comparison with the younger cohorts), while both the percentages (*P *= 0.010) and absolute numbers (*P *= 0.016) of CD8+ cells co-expressing CD49d were significantly lower in the older group compared to the younger cohorts (Table 2). The expression of CD49d on CD4+ and CD8+ T-cells was further assessed as the proportion of cells among each sub-set co-expressing the integrin. Among the older adults, 70.6% of CD4+ cells co-expressed CD49d, compared to 86 - 90% co-expression in the younger cohorts (*P *= 0.001). Also among the older adults, 93% of CD8+ cells co-expressed CD49d, compared to 98 - 100% co-expression in the younger cohorts (*P *= 0.009). Levels of plasma interferons (IFN-α and IFN-β2) did not differ significantly between the cohorts assessed in this study (Table 3).

**Table 3:** Plasma interferon levels (mean ± SEM [upper and lower range values in parentheses])

Plasma cytokine (pg/mL)	Young adults		Older adults	*P*
	Non-exercising	Exercising		
IFN-α	97.2 ± 25.0 (3.2-213.8)	102.7 ± 33.2 (0 - 284.9)	106.0 ± 43.4 (0-322.0)	*0.959*
				
IFN-β2	21.3 ± 6.1 (0.4-85.7)	14.0 ± 6.9 (0-72.4)	9.0 ± 2.3 (0-30.7)	*0.574*

IFN = Interferon.

It is recognised that the number of subjects participating in these studies was small; however the results merit consideration in future investigations of immunosenescence and the impact on the health of older adults. The results for absolute T-cell numbers indicate turnover rates were maintained in this cohort of older adults [8]; while the significant cohort differences in the percentage of CD4 and CD8 T-cells observed here are likely to reflect an age-related shift in percentage of cells expressing a phenotype, as typically happens during immunosenescence [1]. Age-related losses occur in the diversity of CD8 T-cells and are accompanied by a reducing pool of naïve CD8 T-cells [8].

Reduced expression of CD49d on T-cells, in particular as CD4+ and CD8+ sub-sets of CD3+ cells, in the older adults, indicates potentially impaired capability of T-cells to interact with HEVs and APCs. Therefore the capability of naïve T-cells to circulate through lymph nodes could be affected which would impact on T-cell activation and reaction to novel antigenic exposure. In rodent models, it has been shown that the ability to upregulate αβ integrin chains in response to stimulus is impaired in older mice [21] which consequently diminishes their resistance to bacterial infection; hence, it is also possible that reduced α4 integrin expression in older humans could contribute to their increased infection rates. In contrast, there were no differences between the young non-exercising and exercising adults for lymphocyte expression of α4 integrin so for these cohorts there are no apparent exercise induced changes to this immune parameter; hence, this study suggests that exercise-related physiological stress may not affect integrin expression, although the mechanisms influencing exercise induced T-cell redistribution are still to be determined [26]. Type I interferons have been reported to regulate expression of cell adhesion molecules [13,14], so an additional parameter assessed in this study was to measure circulating levels of IFNα and IFNβ2; however, we found no statistical evidence of a concurrent reduction in blood interferon levels among the older subjects that might have been contributory towards their reduced T-cell expression of α4 integrins.

It is possible that the age- related changes to α4 integrin expression on T-cells noticed in these older adults are an early indicator of loss of homeostatic mechanisms. While functional data on naïve CD4 T-cells from individuals between the ages of 60-70 indicate that these cells are fully competent and do not have proliferative defects, the homeostatic mechanisms that maintain levels of naïve T-cells eventually fail after the ages of 70-75 years [8]. Hence, the recording here of reduced α4 expression may precede and contribute to the known senescence of functional immune responses that are reliant on lymphocyte trafficking to peripheral sites, such as delayed-type hypersensitivity response [27].

While no further evidence can be provided here regarding the molecular or physiological basis underlying a reduction in CD49d expression on T-cells in older adults, these results in combination with prior knowledge (that immunosenescence in the T-cell compartment is characterised by the presence of fewer antigen-naïve cells and a reduced TCR repertoire), may lead to a better understanding of how immune competence is affected by ageing.

## Abbreviations

APC: antigen presenting cell; HEV: high endothelial venule; FACS: fluorescence activated cell sorter; IFN: interferon; TCR: T-cell receptor

## Competing interests

The authors declare that they have no competing interests.

## Authors' contributions

CC, MC and CW designed and planned the research. CC recruited participants, collected data, co-ordinated blood collection and full blood count analysis testing, and performed cytokine analysis. CC, MC, CW interpreted results, co-wrote paper, and read and approved this version of the manuscript.
